# Specific Disease Knowledge as Predictor of Susceptibility to Availability Bias in Diagnostic Reasoning: a Randomized Controlled Experiment

**DOI:** 10.1007/s11606-020-06182-6

**Published:** 2020-09-15

**Authors:** Sílvia Mamede, Marco Goeijenbier, Stephanie C. E. Schuit, Marco Antonio de Carvalho Filho, Justine Staal, Laura Zwaan, Henk G. Schmidt

**Affiliations:** 1 Institute of Medical Education Research Rotterdam, Erasmus MC, University Medical Centre Rotterdam, Rotterdam, The Netherlands; 2 Department of Psychology, Education & Child Studies, Erasmus University Rotterdam, Rotterdam, The Netherlands; 3 Department of Internal Medicine, Erasmus Medical Center, Rotterdam, The Netherlands; 4 Centre for Educational Research and Development in Health Professions, University Medical Centre, Groningen, The Netherlands; 5 Internal Medicine Department, School of Medical Sciences, State University of Campinas, Campinas, Brazil

**Keywords:** diagnostic error, availability bias, diagnostic reasoning, clinical knowledge

## Abstract

**Background:**

Bias in reasoning rather than knowledge gaps has been identified as the origin of most diagnostic errors. However, the role of knowledge in counteracting bias is unclear.

**Objective:**

To examine whether knowledge of discriminating features (findings that discriminate between look-alike diseases) predicts susceptibility to bias.

**Design:**

Three-phase randomized experiment. Phase 1 (bias-inducing): Participants were exposed to a set of clinical cases (either hepatitis-IBD or AMI-encephalopathy). Phase 2 (diagnosis): All participants diagnosed the same cases; 4 resembled hepatitis-IBD, 4 AMI-encephalopathy (but all with different diagnoses). Availability bias was expected in the 4 cases similar to those encountered in phase 1. Phase 3 (knowledge evaluation): For each disease, participants decided (max. 2 s) which of 24 findings was associated with the disease. Accuracy of decisions on discriminating features, taken as a measure of knowledge, was expected to predict susceptibility to bias.

**Participants:**

Internal medicine residents at Erasmus MC, Netherlands.

**Main Measures:**

The frequency with which higher-knowledge and lower-knowledge physicians gave biased diagnoses based on phase 1 exposure (range 0–4). Time to diagnose was also measured.

**Key Results:**

Sixty-two physicians participated. Higher-knowledge physicians yielded to availability bias less often than lower-knowledge physicians (0.35 vs 0.97; *p* = 0.001; difference, 0.62 [95% CI, 0.28–0.95]). Whereas lower-knowledge physicians tended to make more of these errors on subjected-to-bias than on not-subjected-to-bias cases (*p* = 0.06; difference, 0.35 [CI, − 0.02–0.73]), higher-knowledge physicians resisted the bias (*p* = 0.28). Both groups spent more time to diagnose subjected-to-bias than not-subjected-to-bias cases (*p* = 0.04), without differences between groups.

**Conclusions:**

Knowledge of features that discriminate between look-alike diseases reduced susceptibility to bias in a simulated setting. Reflecting further may be required to overcome bias, but succeeding depends on having the appropriate knowledge. Future research should examine whether the findings apply to real practice and to more experienced physicians.

**Electronic supplementary material:**

The online version of this article (10.1007/s11606-020-06182-6) contains supplementary material, which is available to authorized users.

## INTRODUCTION

A National Academy of Medicine report recently emphasized that diagnostic errors may be one of the most common and most harmful of patient safety problems.^1^ Retrospective studies have implicated cognitive factors in around three-quarters of real-life diagnostic errors.^2–4^ The sources of these “cognitive errors” have been much discussed.

Many authors attribute cognitive errors primarily to flaws in reasoning process. Behind most flaws would be biases induced by heuristics^5^ routinely used by physicians to make fast, intuitive judgments.^6–9^ For instance, physicians tend to focus on considering diagnoses that are more easily retrievable from memory. Though efficient, this may lead to “availability bias” when what comes more easily to mind is an incorrect diagnosis.^10, 11^ This viewpoint seems supported by retrospective studies. For example, an investigation of 100 cases of diagnostic errors in academic hospitals attributed only around 3% of them to knowledge deficits.^2^ The vast majority of errors were classified as flaws in the physician’s reasoning such as overestimation of the usefulness of a clinical finding (e.g., wrong diagnosis of sepsis in a patient with stable leukocytosis in the setting of myelodysplastic syndrome).^2^

Conversely, other authors have shown the difficulty of retrospectively identifying biases^12^ and argued that the literature may have underestimated the role of knowledge deficits in diagnostic error.^13, 14^ Because they influence how the physician reasons through the case, disentangling between processing and knowledge in the chain of causation would be hardly possible.

Indeed, studies such as the aforementioned review^2^ provide much valuable insights into *what* went wrong in the physicians’ reasoning, but *why* the failure occurred is actually uncertain.^14^ It may well be that instead of *either* knowledge deficits *or* processing bias, the interplay between the two is behind most errors. For example, if the physician was aware of myelodysplastic syndrome but did not know that it could present itself in a particular way, knowledge of the syndrome would probably not be activated to help overcome the influence of the salient findings that led to the wrong diagnosis. Activation would depend on specific features of the knowledge of myelodysplastic syndrome as represented in the physician’s memory, for instance, the variety of findings associated with the disease and the storage of critical diagnostic cues. This idea is supported by psychological research.^15, 16^ Nevertheless, whether knowledge counteracts bias remains controversial. Investigating whether it does requires measurements that capture specific differences in physicians’ knowledge. To our knowledge, these measurements have not yet been used to investigate the diagnostic error.

The present study aimed to examine the interplay between knowledge deficits and processing bias in the origin of diagnostic error. Physicians diagnosed cases under conditions that tend to induce availability bias. We measured physicians’ specific disease knowledge and, by taking time spent in diagnosis as an indication of reasoning mode (assuming intuitive reasoning to require less time), we examined the contribution of content knowledge and reasoning process in counteracting bias. We expected specific disease knowledge to be the primary predictor of susceptibility to bias, with physicians with more knowledge resisting bias more frequently possibly independent of diagnosis time.

## METHODS

### Study Design

**Figure 1:**
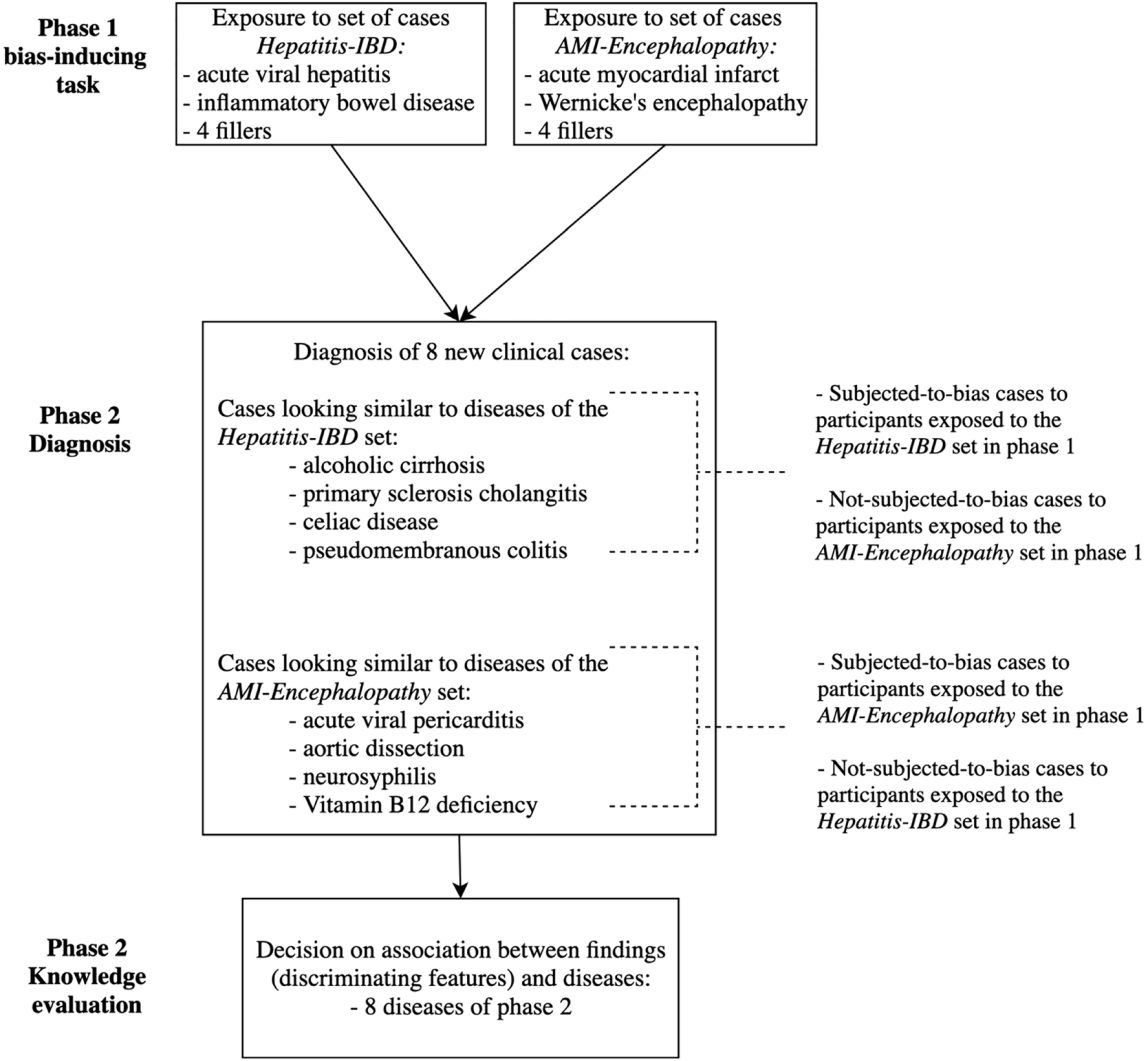
Study design.

The experiment consisted of three phases, presented to participants as independent, unrelated studies (see Fig. 1). In phase 1 (bias-inducing), participants evaluated the plausibility of a diagnosis given for clinical cases from one of two case sets (either “hepatitis-IBD,” containing acute viral hepatitis and inflammatory bowel disease, or “AMI-encephalopathy,” containing acute myocardial infarction and Wernicke’s encephalopathy). In phase 2 (diagnosis), all participants diagnosed the same new cases, 4 resembling diseases of the hepatitis-IBD set, 4 resembling diseases of the AMI-encephalopathy set (but all with different diagnoses). In phase 3 (knowledge evaluation), all participants decided whether a particular symptom is associated with a particular disease or not. Accuracy of participants’ decisions assessed the breadth and strength of the associations between the disease and its clinical findings as stored in memory.

Previous exposure to a disease that looks like the case at hand was expected to induce availability bias, causing diagnostic errors in the 4 cases of phase 2 that resembled the ones encountered in phase 1. Notice that all participants diagnosed 4 cases that were subjected to the bias-inducing treatment and 4 cases that were not, but which cases fell into each category depended on the diseases encountered in phase 1. (A similar procedure induced availability bias in previous studies.)^10, 17^ Physicians who performed better in phase 3 were expected to resist availability bias more frequently.

### Participants

All Erasmus MC internal medicine residents who had at least 1 year of clinical practice were invited by two co-authors (M.G.; S.S.) for the study. All participants provided written consent. The DPECS/Erasmus University Rotterdam Research Ethics Review Committee approved the study. Supplement 1 provides additional information on participants.

### Materials and Procedure

Sixteen written clinical cases were used in phases 1 and 2.^1^ The cases were developed by board-certified internists, validated, and used in previous studies with similar participants.^18, 19^ We selected cases at an intermediate level of difficulty to allow room for errors, without manipulating the cases to make them particularly bias-prone. Supplement 2 presents a sample case.

In phase 1, participants were randomly assigned to receive either the hepatitis-IBD or the AMI-encephalopathy set. Each set contained, besides 4 fillers, 2 cases of interest for phase 2 (see below). Each case included a suggested diagnosis, and the participant was requested to rate the likelihood that it was correct.

In phase 2, all participants diagnosed the same 8 new cases, 4 of them resembling diseases of the hepatitis-IBD set, 4 diseases of the AMI-encephalopathy set (Fig. 1). For example, a patient with celiac disease or pseudomembranous colitis may present with manifestations similar to IBD. For each case, participants were asked to type (free-text) the most likely diagnosis doing their best to be accurate and fast.

For the knowledge evaluation (phase 3), three internists (M.G; S.K.; M.C.) prepared, for each disease of phase 2, a list of 24 clinical findings (including medical history, complaints, physical examination, and diagnostic tests) containing 12 “filler” (unrelated) findings and 12 findings associated with the disease. Among the latter, the internists selected findings that are critical to discriminate between usual alternative diagnoses for the disease (hereafter “discriminating features”). Supplement 2 presents a sample disease. Phase 3 presented a “recognition-task” traditionally used in psychology to assess knowledge as stored in one’s memory.^20, 21^ We used this task to categorize physicians’ knowledge because it measures *specific* disease knowledge, which cannot be accurately inferred from variables such as years of practice. Participants decided as fast as possible whether a symptom is associated with a disease or not by pressing a keyboard key (Supplement 1 presents additional information). For each disease, the first screen presented the name of the disease and the subsequent screens presented, one by one, the 24 findings. The order of presentation of the diseases and of the findings for each disease was randomized.

After the three phases, the participants answered questions on demographic information, clinical experience, and two probing questions. Finally, for feedback, they saw the cases with the correct diagnosis.

All phases were carried out sequentially in a single session, by using Qualtrics, an online survey platform that automatically registers participants’ responses and response time.

### Outcome Measurements

The main outcome measure was the frequency with which the phase-1 diseases were given as the diagnosis of similar-looking cases in phase 2, e.g., IBD on the cases of celiac disease and pseudomembranous colitis. Notice that these two cases could eventually be incorrectly diagnosed as IBD even if the physician was not under the influence of availability bias. However, errors would probably have been induced by the bias if their frequency increased among physicians who encountered IBD in phase 1 relative to those who did not.

Diagnosis time (automatically registered by the program) was taken as an indication of how extensively the physician processed the case, with increased time indicating more analytical reasoning.

Participants’ knowledge was measured by performance in phase 3. For each participant, for each disease, we computed the proportion of correct decisions made for the discriminating features. The average for all diseases was obtained, and based on its median, we split participants into two knowledge-level groups.

### Statistical Analysis

We computed the mean frequency with which the phase-1 diagnoses were mentioned in phase 2 (range 0–4) on subjected-to-bias and not-subjected-to-bias cases. We performed a mixed ANOVA with knowledge level as a between-subjects factor (higher-knowledge vs. lower-knowledge) and exposure to bias (subjected-to-bias and not-subjected-to-bias) as a within-subjects factor on the mean frequency of phase-1 diagnoses. This analysis assessed whether diagnostic errors increased due to availability bias and whether stronger knowledge of discriminating features counteracted the bias. Post hoc independent *t* tests compared the frequency of this type of error made by higher-knowledge and lower-knowledge physicians on subjected-to-bias and not-subjected-to-bias cases. Paired *t* tests compared the frequency of errors on subjected-to-bias and not-subjected-to-bias cases within the same knowledge-level group.

A similar ANOVA was performed on the mean time spent to diagnose a case to assess whether subjected-to-bias cases triggered engagement in a more analytical reasoning mode and whether this engagement depended on physicians’ knowledge level. This analysis was relevant because of eventual differences in the frequency of diagnostic errors could be due to different reasoning approaches adopted by the knowledge groups rather than by knowledge itself.

Descriptive statistics were obtained for participants’ age, gender, years of clinical experience, and mean ratings of experience (range 0–5) with the diseases of the study, and we checked for differences between higher-knowledge and lower-knowledge groups (see Supplement 1).

All analyses were performed in SPSS version 25, with the level of significance set at *p* < 0.05 two-sided.

## RESULTS

Sixty-two (out of 70) participants performed all the tasks according to the instructions and completed the study session. The two knowledge-level groups did not significantly differ in background characteristics, or clinical experience (Table 1). Supplement 1 provides additional information.

**Table 1:** Participants’ Characteristics

	Lower knowledge level (*N* = 31)	Higher knowledge level (N = 31)	Overall (*N* = 62)
Age (years)			
Mean (95% CI)	30.90 (29.97–31.82)	30.84 (29.99–31.69)	30.87 (30.26–31.47)
Sex			
Male	7 (23%)	14 (45%)	21 (34%)
Female	23 (77%)	17 (55%)	40 (66%)
Number of years in clinical practice			
Mean (95% CI)	3.27 (2.61–3.93)	3.53 (2.81–4.26)	3.41 (2.93–3.89)
Experience with the diseases of the study (range 0–5)			
Mean (95% CI)	2.41 (2.25–2.56)	2.43 (2.26–2.61)	2.42 2.42 (2.31–2.54)

**Figure 2:**
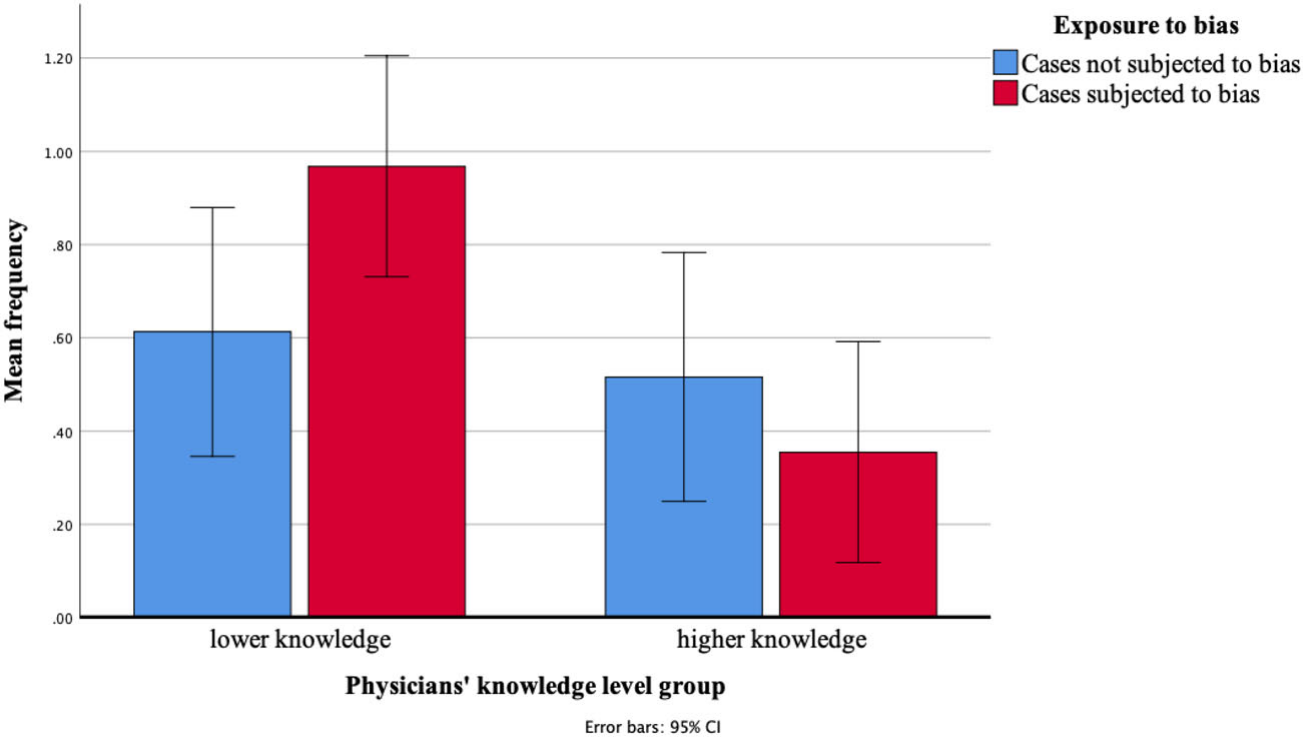
Frequency of diagnoses of phase 1 given to similar-looking cases in phase 2 (range 0–4) as a function of exposure to bias and knowledge level.

Figure 2 presents the frequency with which phase-1 diagnoses were incorrectly given to similar-looking cases in phase 2. Overall, the frequency did not differ between subjected-to-bias and not-subjected-to-bias cases (*p* = 0.43). As expected, overall, lower-knowledge physicians made these errors more frequently than physicians with a stronger knowledge of discriminating features (*p* = 0.01). A significant interaction effect was found (*p* = 0.03) between knowledge level and exposure to bias. Higher-knowledge and lower-knowledge physicians only differed in how frequently they mentioned phase-1 diagnoses in phase 2 on subjected-to-bias cases. On these cases, higher-knowledge physicians yielded to availability bias, i.e., confused the cases with the previously seen diseases, less often than physicians from the lower-knowledge group (*p* = 0.001). When the cases were not subjected to bias, the frequency of phase-1 diagnoses did not significantly differ between higher-knowledge and lower-knowledge physicians (*p* = 0.60). Within-group analysis showed that lower-knowledge physicians tended to make more of these errors on subjected-to-bias compared with that on not-subjected-to-bias cases (*p* = 0.06). Conversely, the frequency of errors was not affected by exposure to bias among higher-knowledge physicians (*p* = 0.28). Table 2 presents all comparisons.

**Table 2:** Synthesis of the Comparisons Made and Findings Obtained

	Overall	Subject to bias cases	Not subject to bias cases	*Comparisons overall and within group: absolute difference (95% CI); p value*
Frequency of phase-1 diagnoses in phase 2				
Overall		0.66	0.56	0.10 (− 0.34 to 0.14); *p* = 0.43
Lower-knowledge group	1.58	0.97	0.61	0.35 (− 0.02 to 0.73); *p* = 0.06
Higher-knowledge group	0.87	0.35	0.52	− 0.16 (− 0.46 to 0.14); *p* = 0.28
*Comparisons overall and between groups: absolute difference (95% CI); p value*	0.71 (0.17 to 1.25); *p* = 0.01	0.62 (0.28 to 0.95); *p* = 0.001	0.10 (− 0.28 to 0.47); *p* = 0.60	
Mean time spent to diagnose (seconds)				
Overall		79.42	75.64	3.78 (0.23 to 7.33) *p* = 0.04
Lower-knowledge group	76.77	79.16	74.38	
Higher-knowledge group	78.29	79.67	76.90	
*Comparisons overall and between groups: absolute difference (95% CI); p value*	1.51 (− 8.64 to 5.62); *p* = 0.67	0.51 (− 7.57 to 8.59); *p* = 0.90	2.51(− 5.34 to 10.37); *p* = 0.52	

**Figure 3:**
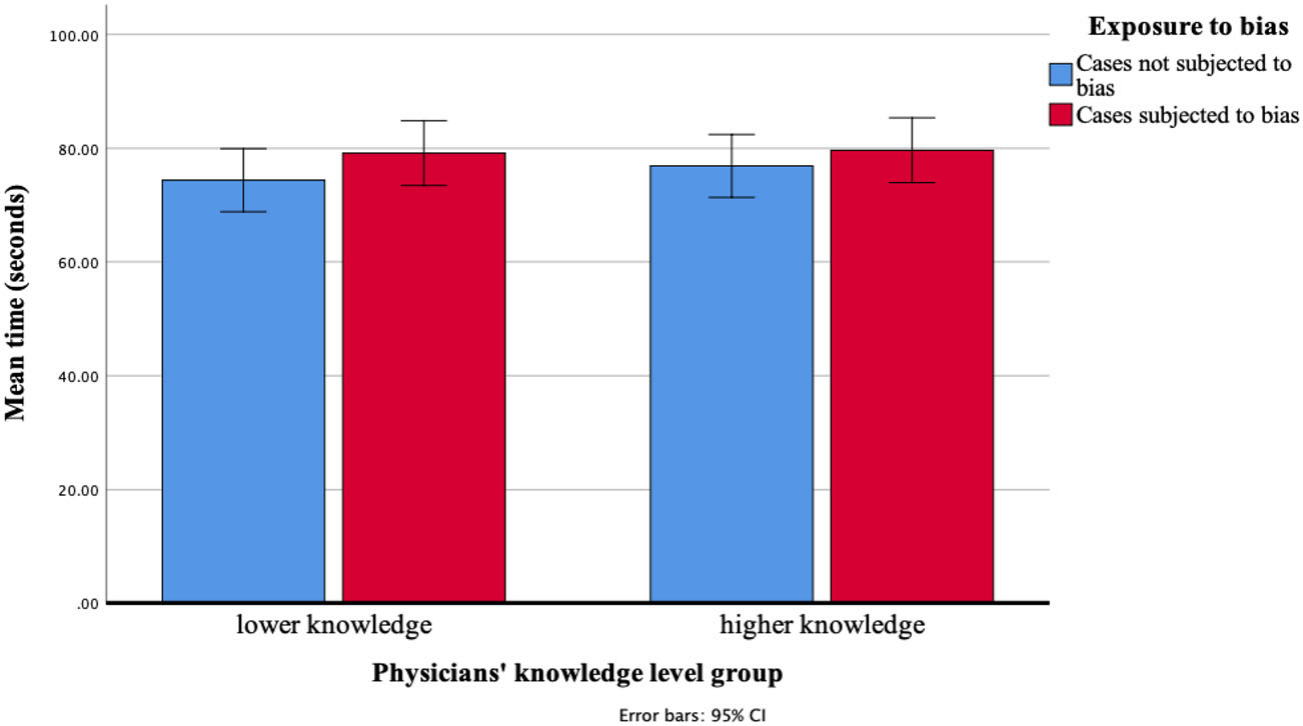
Time spent in diagnosing a case as a function of exposure to bias and knowledge level.

Figure 3 presents the mean time (in seconds) spent diagnosing a case. Overall, physicians took more time to diagnose the cases when they were preceded by a similar-looking disease (subjected-to-bias) than when they were not (not-subjected-to-bias) (*p* = 0.04). Higher-knowledge and lower-knowledge physicians did not differ in diagnosis time either on subjected-to-bias (*p* = 0.90) or in not-subjected-to-bias cases (*p* = 0.52). (See Table 2).

## DISCUSSION

In this experiment, differences in knowledge of clinical features that discriminate between similar-looking diseases predicted susceptibility to bias among physicians with a similar level of training and clinical experience. To capture diagnostic errors actually induced by availability bias, we measured the frequency with which physicians gave a case a similar (but incorrect) diagnosis after having (or not) recently encountered that diagnosis in a look-alike case. This type of error attributable to availability bias increased by 58% among physicians with less knowledge of discriminating features. Conversely, the performance of physicians with stronger knowledge was unaffected. The similar time spent by the two groups of physicians to diagnose subjected-to-bias cases suggests that neither group engaged more extensively in analytical reasoning. Rather than differences in reasoning mode, differences in specific disease knowledge seem therefore to explain the variation in susceptibility to bias. Interestingly, regardless of knowledge level, physicians spent significantly more time to diagnose the same cases when they were subjected to bias than when they were not.

At first glance, the finding that more knowledgeable physicians are less susceptible to bias seems like plain common sense. However, evidence on the role of knowledge in counteracting bias is contradictory. Whereas some psychology studies suggested domain-specific knowledge to help,^22, 23^ others found experts and novices to be equally vulnerable to biases.^24^ The medical literature reports a slight negative relationship between diagnostic accuracy and experience in clinical practice, which is a proxy for (experiential) knowledge.^25, 26^ Moreover, investigation of real-life diagnostic errors often identified bias-induced faulty reasoning as a primary driver, without underlying knowledge deficits.^2–4^ Whether the amount of disease knowledge per se explains sensitivity to bias seems therefore unclear.

The present study measured not knowledge in general but specific features of disease knowledge: the breadth, accuracy, and strength of associations between the disease and critical diagnostic cues. The findings suggest that this specific knowledge that some physicians had while others not predicts susceptibility to bias. The participants reported a similar number of years in practice and clinical experience. Nevertheless, because experiences throughout education and practice differ across physicians, differences in disease knowledge as stored in the physicians’ memory are unavoidable. When contextual cues, such as a similar-looking disease, direct physicians’ attention to findings in the case that are in fact irrelevant, a wrong diagnostic hypothesis may be generated. Recognition of findings that are actually more relevant can trigger reasoning restructuring. Strong knowledge of critical diagnostic findings would make this recognition more likely, consequently increasing resistance to bias. A recent study by our group showed an intervention to increase physicians’ disease knowledge to reduce susceptibility to bias.^17^ The intervention emphasized discriminating features but probably resulted in overall refinement of mental representations of diseases, which may have explained its benefits. The present study goes a step further, focusing on the role of knowledge of discriminating features in counteracting bias.

Educating physicians on circumstances that require a more analytical reasoning approach has been often suggested as a strategy to reduce bias-induced diagnostic errors.^27, 28^ However, without such training, our participants took more time to diagnose the *same* cases when the cases were subjected to bias than when they were not. We did not examine how physicians used this time, and the difference is small. Nevertheless, it suggests that the bias-inducing circumstance per se triggered a more thorough analysis. Physicians from both groups apparently “struggled” to overcome the influence of the bias. Recent psychological research suggests that most people unconsciously detect the risk of bias, but whereas some succeed to inhibit the bias-induced response, others fail.^29, 30^ A bias-induced hypothesis may be hard to reject. It is easily retrieved, looks reasonable and, after generated, influences interpretation of other findings.^31^ A previous study demonstrated that deliberate reflection upon initial diagnoses tended to counteract availability bias.^10^ The present study reveals that benefitting from a more thorough analysis to actually succeed in overcoming bias apparently depends on more robust knowledge of critical diagnostic cues.

These findings have implications for the education of students and physicians. Clinical teaching usually addresses differential diagnosis, but emphasizing discrimination between look-alike diseases seems justifiable. This requires effective educational strategies for fine-tuning mental representations of diseases, probably stratified by the learners’ training level.^32^ The development of these strategies should receive more attention within research on how to reduce bias-induced errors. This research has focused on approaches to improving the reasoning process per se which have hitherto shown little benefits.^14, 33, 34^

The study has limitations. First, all phases occurred in a single session, with the knowledge evaluation task placed after the diagnosis to avoid priming. Activation of knowledge during the diagnosis may have influenced performance in the subsequent task. Second, the study was conducted in a simulated setting with written clinical cases, and the effect of the bias on accuracy was relatively small (noteworthy, the bias-inducing “treatment” was subtle). Whether bias would be more or less harmful in real practice is unclear. In real encounters, physicians could access other sources of knowledge or benefit from other cues. On the other hand, they are subject to the negative influence of wrong initial impressions on subsequent information gathering,^31^ while written cases provide all information required for the diagnosis. Third, our participants had few years of clinical practice and medium experience with the diseases of the study. The findings may not generalize to more experienced physicians though evidence hitherto does not show experience per se to decrease susceptibility to bias.^35^ Fourth, we studied availability bias, and other biases such as affective bias may be less influenced by knowledge. Finally, we used diagnosis time to assess how extensively physicians processed the case, without further examining processing. While reflection requires time, time per se does not guarantee that reflection occurred.

Future research should examine the mechanisms through which knowledge of discriminating features helps counteract bias. This may guide the development of educational strategies for the refinement of disease knowledge. Further research should also explore the role of knowledge in counteracting other types of cognitive bias besides availability bias and among more experienced physicians.

Summing up, physicians with stronger knowledge of clinical features that discriminate between similar-looking diseases made fewer diagnostic errors caused by availability bias than their peers with less of such knowledge. The increased resistance to bias occurred despite the similar amount of time spent in diagnosis, with both groups taking longer to diagnose subjected-to-bias than not-subjected-to-bias cases. Taken together, these findings suggest that specific disease knowledge predicts susceptibility to bias. Reflecting further about the case may be required to overcome the influence of bias, but succeeding or not depends on having the appropriate knowledge to bring to the task.

## Electronic Supplementary Material

ESM 1 (DOCX 26 kb)

## Footnotes

1 Twelve cases were actually relevant to the study, while four cases were “fillers,” used only to disguise the study purpose and not included in the analysis.
