# Supplementary material for: Specific Disease Knowledge as Predictor of Susceptibility to Availability Bias in Diagnostic Reasoning: a Randomized Controlled Experiment
Source: J Gen Intern Med. 2020 Sep 15;36(3):640–6. doi: 10.1007/s11606-020-06182-6 (PMC7947124; doi:10.1007/s11606-020-06182-6)
Supplement: Supplementary file 1 — (DOCX 26 kb) [file 11606_2020_6182_MOESM1_ESM.docx]

**Supplementary Material**

**Supplement 1**

**Additional information on participants’ recruitment and results**

**Sample size estimation**

A priori power analysis was performed based on findings of a previous study on bias in diagnostic reasoning with similar participants.^18^ Assuming a Cohen’s *f* of 0.27 as the to-be-detected effect size, and the standard alpha level of 0.05, the analysis indicated that a sample size of 66 participants would be sufficient to achieve a power of 0.80.

**Recruitment**

All physicians in training to become specialists in internal medicine at the ErasmusMC were invited by email by two co-authors (M.G.; S.K.) to participate in the study which would take place during lunch time on a monthly educational meeting. Dutch academic hospitals use to offer two different possibilities for such training: a position that is officially recognized as residency and a position that is not formally a residency program but allows for similar training. Physicians from both positions were invited, but a minimum of one year of clinical experience was established as a criterion for eligibility to ensuring that only physicians with some clinical experience would participate in the study. As an acknowledgment for their participation, each participant received a compensation of € 50,00, previously agreed to be donated to a residents’ scientific meeting.

**Exploratory analysis and probe questions**

After all the study phases, the participants responded two probing questions, the first on the study purpose and research questions (to verify whether participants had recognized the attempt to induce bias), and the second on how they had understood and engaged with the tasks.

Regarding the first question, none of the participants reported to have suspected that the study tasks belonged to the same study or that they could induce bias or cause errors. However, on the second question, 5 out of the 67 participants who started the session reported troubles such as disturbances or distraction by external demands while working on the tasks. An exploratory analysis for response times in the diagnostic task showed z-scores greater than 2.5 for these 5 participants, and they were therefore excluded from the analysis.

**Additional information on statistical analysis and results**

Sixty-two out of the 70 invited physicians accepted the invitation and completed the study tasks. Training year ranged from 1 to 5, with median of 3 years. We examined whether the higher-knowledge and the lower-knowledge group differed in background variables that tend to influence clinical performance to exclude that differences in susceptibility to bias could be explained by differences in these characteristics rather than in knowledge. Separate one-way ANOVAs with knowledge level as between-subjects factor were performed to compare the two groups regarding age, number of years in clinical practice, training year, and self-reported experience with the diseases of the study. These tests showed that the groups did not differ in age (*p* = 0.92), number of years in clinical practice *(p* = 0.58), training year (*p* = 0.61), or experience with the diseases of the study (*p* = 0.77). Additionally, we examined whether the two knowledge groups were equally distributed across the two bias-inducing conditions in phase 1 (set A or B). A Fisher’s Exact test showed that the number of participants from the two knowledge groups who were biased towards each phase-1 set did not significantly differ (*p* = 0.20; two-tailed).

The main outcome measurement of the study was the frequency with which the phase-1 diseases were mistakenly mentioned as the diagnosis of similar-looking cases in phase 2. To count these diagnoses two co-authors (M.C.; S.M.) independently and blindly counted the phase-1 diagnoses among the responses given by the participants in phase 2. Agreement was near perfect (ICC = 0.97), and discrepancies settled by discussion. Although identifying the phase-1 diagnoses in the participants’ responses was a straightforward task, it can always happen that participants use different wording to refer to a diagnosis. This may raise doubts about whether a response indicates or not the same diagnosis of the bias-inducing case (for example, ‘thiamine deficiency’ after exposure to the case of Wernicke’s encephalopathy). Having two raters independently and blindly counting the diagnoses and ensuring that only agreed upon responses are considered addresses this issue.

**Supplement 2**

**Example of a clinical case used in the study**

A 33-year-old Thai woman, who lives in the Netherlands for the last 27 years, reports having observed jaundice for several weeks. It was initially episodic, but has become constant in the last week. She also complains of severe itching, but there are no noticeable scratches or blue spots. She says she has occasionally felt her body warm, but she did not measure her temperature. She reports mild pain in the right hypochondrium and denies loss of appetite and weight loss.

The patient is married, has two healthy children, and works as a nurse at a local hospital. Her medical history reports ulcerative colitis (she has used mesalazine for 2 years) and an episode of multiple traumatic injuries with a blood transfusion fourteen years ago.

*Physical examination:*

The physical examination shows a not acutely ill, icteric patient. Normal hemodynamic and respiratory conditions. Head/neck: without abnormalities, except icterus. Abdomen: mild pain on palpation of the hypochondrium, without other abnormalities. Extremities: without particularities.

*Diagnostic tests (normal ranges into brackets):*

Hemoglobin, 117 g/L (120-140 g/dL); Platelets, 200 x 10^9^/L (150-300 x 10^9^/L); Leukocytes, 14 x10^9^/L (3,5-10 x 10^9^/L) with normal differential; Conjugated bilirubin, 5.02 mg/dL (at the primary health care doctor, 6.84 mg/dL) (<0.6 mg/dL); GGT, 123 U/L (<38 U/L); AF, 560 U/L (< 98 U/L); AST, 234 U/L (<31 U/L); Albumin, 29 g/L (35-50 g/L); Renal function and electrolytes, normal; Calcium, 7.92 mg/dL; (8.8-10.6 mg/dL); p-ANCA, positive; Anti-mitochondria antibodies (M2 fraction), negative; urinalysis, no particularities.

Abdomen ultrasonography: enlarged liver; biliary tract with caliber within normal range, but without completely normal aspect. There is no hyperechogenicity, no visible glands. MRCP is recommended.

*Diagnosis:* Primary sclerosing cholangitis

**Example of a disease with the clinical findings used in the knowledge evaluation task (phase 3)**

**Primary sclerosis cholangitis**

(Discriminating features underlined)

| Related findings | Unrelated findings |
| --- | --- |
| Fatigue  Characteristic layered fibrosis (onion skin fibrosis)  Colic pain  Hepatomegaly  Enlarged gallbladder  Dark urine  Increased fat in faeces  Portal hypertension  Pyoderma gangraenosum  Recurrent cholangitis  Arthritis  Specific cholangiogram with ductus scarring/strictures | Enlarged mediastinum  Cardiac tamponade  Pulmonary edema  Cerebellar ataxia  Orthostatic hypertension  Hypernatremia  Increased troponin  EKG with ST segment elevation  Dry cough  Hemiparesis  recurrent headache  hematuria |
